# A cross-sectional study on Saudi pharmacists working as medical representatives: What attracted them and what is keeping them in this sector—Misconceptions and reality

**DOI:** 10.3389/fpubh.2023.996536

**Published:** 2023-03-08

**Authors:** Dalia Almaghaslah, Abdulrhman Alsayari

**Affiliations:** ^1^Department of Clinical Pharmacy, College of Pharmacy, King Khalid University, Abha, Saudi Arabia; ^2^Department of Pharmacognosy, College of Pharmacy, King Khalid University, Abha, Saudi Arabia

**Keywords:** medical representatives, Saudi pharmacists, misconceptions, reality (“réel”), cross-sectional study

## Abstract

**Background:**

The government in Saudi Arabia issued a labor reform initiative to renationalize the pharmacy profession in pharmaceutical companies to generate more employment for Saudi pharmacists. Considering the nationalization of the pharmacy workforce employed in this sector, as well as the pharmacists' preferences toward working in this setting, the current study was conducted determine the reasons to choose this career pathway, to clarify the common misconceptions about this sector and to assess job satisfaction, work commitment, and intentions to leave.

**Methods:**

An online self-administered questionnaire was used to collect data from pharmacists working as medical representatives across Saudi Arabia. A total of 133 medical representatives participated in the study.

**Results:**

The main factors that motivated study participants to join this sector included performing a socially important job, receiving a high salary, and further career development. The common misconceptions about the sector, such as lacking honor and value and considering commercial values to be accepted, were found to be incorrect by the medical representatives. A high job satisfaction level, high work commitment, and low intentions to leave the sector were reported by participants.

**Conclusion:**

Working as a medical representative in a pharmaceutical company is an appealing career choice that fulfills pharmacists' career ambitions and may help in creating more jobs for the increasing numbers of pharmacy graduates.

## Introduction

The pharmacist's role has evolved enormously over the past two decades (1). The traditional role of dispensing medication has expanded into more knowledge utilization in various settings (1). Pharmacy career pathways now extend beyond the product-oriented functions of dispensing and distributing medications and pharmaceutical products to more advanced opportunities, such as health informatics, patient safety, and public health (1, 2). The pharmaceutical industry is an area that allows pharmacists to practice as a medicine expert.

A medical representative is defined as “a business staff person of pharmaceutical enterprises, who acts on behalf of the enterprise and is responsible for contact with medical personnel, providing relevant information about the quality, effectiveness and safety of drugs, as well as collecting and disseminating information” (3, 4).

The pharmacy professional in Saudi Arabia has radically evolved over the last decade. Historically, different pharmacy sectors in the country were mainly run by non-indigenous pharmacy personnel due to the limited numbers of Saudi pharmacists (5, 6). For instance, pharmaceutical companies are the second largest employment sector in the field, following community pharmacies, employing 28.27% of the total pharmacy workforce. However, 2017 data from the Saudi Commission for Health Specialties (SCFHS) showed that only 11.1% of the total employees were Saudi nationals.

Employment rules and regulations regarding renationalizing the profession have been issued by the government as a response to the increasing number of national pharmacy graduates (7). Nationalizing the profession began by gradually replacing non-indigenous staff working in hospital pharmacies, the sector most preferred by national pharmacists; this sector is currently almost completely Saudised (7). Similar regulations were recently imposed on the largest employment sectors, community pharmacy and the pharmaceutical industry. In 2018, The Saudi Food and Drug Authority issued a policy outlining the expected rates of Saudising the pharmaceutical companies-−40% by the end of 2019, 80% by 2020 and 100% by 2021 (8). In 2020, community pharmacies began to be renationalized in two stages: 20% by 2020 and 30% by 2021 (9).

Recent local studies looking at pharmacy students' career choices indicated that working in the pharmaceutical industry was among the most favorite sectors (7, 10–12). However, older studies listed the pharmaceutical industry among the least preferred career pathways, indicating a change in pharmacists' attitudes toward the pharmaceutical industry (13, 14). On the other hand, international studies have suggested that medical representatives were generally satisfied with their jobs. However, some aspects of the work were less satisfying, including lack of promotions and benefits, high workload, and job security (15, 16).

Until now, no studies have been conducted on the pharmaceutical industry as a career pathway in Saudi Arabia, especially concerning job satisfaction and work commitment. Published literature that involved the medical representative evaluated physician's acceptance of pharmaceutical gifts and promotions. Studies have raised ethical concerns about drug promotions and marketing (17–20).

Considering the nationalization of the pharmacy workforce employed in this sector, as well as the pharmacists' preferences toward working in this setting, the current study was conducted to find out the reasons for choosing this career pathway, to clarify the common misconceptions about this sector, and to assess job satisfaction, work commitment, and intentions to leave.

## Methods

### Study design

The study used a cross-sectional design. An online self-administered questionnaire was used to collect data from pharmacists working as medical representatives across Saudi Arabia.

### Population and setting

This investigation was carried out with registered pharmacists practicing in national and international pharmaceutical companies in Saudi Arabia. The total number of licensed pharmacists practicing in this sector is 6,896, of which 504 (11.1%) were Saudi in 2018 (6).

### Sample size and sampling procedure

The sample size was determined based on the number of Saudi pharmacists practicing in pharmaceutical companies (504) and calculated by using a Raosoft sample size calculator (http://www.raosoft.com/samplesize.html) with a predetermined margin of error of 5% and a confidence level of 90%. In order to assure study reliability and to minimize erroneous results, the target sample size was set at 177 participants. A non-probability convenience sampling method was selected, including five of the largest national and international pharmaceutical companies in the country. An invitation letter was officially sent to these companies through email. Efforts to maximize the response rate were made by sending multiple reminders emails to the potential participants. A total of 133 completed questionnaires were received, giving a response rate of 75.14%.

### Data collection form

The structured questionnaire was adapted from previous studies (4, 7, 21–25). The questionnaire consisted of 4 domains: demographics/background information, factors for choosing to work in this sector, medical representative's opinions on common perceptions of the sector, job satisfaction, work commitment, and intentions to leave. The questionnaire was piloted with 10 medical representatives who were representative of the study population to determine the clarity of the language and the questionnaire structure. The results of the pilot study are not included in the results. The data collection tool was reviewed and modified based on the feedback received in the pilot. The questionnaire was finalized in the English language in the form of a self-administered online questionnaire. There were no partially completed questionnaire as all questions were fully answered by respondents.

### Inclusion criteria

Participants were expected to have a valid license to practice pharmacy in Saudi Arabia, and being Saudi nationals, are currently working as a medical representative in a pharmaceutical company, have a minimum of a first degree or higher degree in pharmacy, and are willing to take part in the study.

### The exclusion criteria

Those pharmacy personnel practicing in other pharmacy sectors, including hospital pharmacy, community pharmacy, academia, and pharmacy regulation, as well as international medical representatives working in the country, were excluded from the study population.

### Statistical analysis

The collected data were cleared, entered and analyzed by using the Statistical Package for Social Sciences (SPSS) version 26.0 for the Mac. Demographic and background information were described in terms of frequencies. The section titled “factors for choosing to work in this sector” (five items) used a Likert scale ranging from 1 (very unimportant) to 5 (very important), while “perceptions about the medical representative career pathway” (seven items) as well as “job satisfaction, work commitment, and intentions to leave” (five items) used a Likert scale ranging from 1 (strongly disagree) to 5 (strongly agree). Scale items were categorized into themes. The distribution of the scale is presented in percentages, using mean and SD. The internal consistency and reliability of the scales was assessed using Cronbach's alpha coefficient, with the minimum recommended level being 0.70.

### Ethics approval

An ethical clearance was given by The Ethical Committee of the Scientific Research, King Khalid University ECM# 2021-5511. All respondents were asked for their consent before participation in the study.

## Results

Table 1 shows participants' demographics. A total of 133 medical representatives participated in the study. Two thirds (66.9%) of the participants were young pharmacists between the ages of 23 and 30 years, and the majority (86.5%) were male which might indicate that this career path is a male-dominated pharmacy sector that attracts young professionals. The vast majority (90.2%) had achieved the first degree in pharmacy, while just under half (48.1%) had between 1 to 5 years work experience.

**Table 1:** Demographics and background information.

	***n***	**%**
**Age in years**		
23–30	89	66.9
31–40	43	32.3
41–50	1	0.8
>50	0	0
**Gender**		
Male	115	86.5
Female	18	13.5
**Qualification**		
Bachelor degree	120	90.2
Postgraduate degree	13	9.8
**Work experience in years**		
<1	19	14.3
1–5	64	48.1
>5	50	37.6

Table 2 shows the factors considered when choosing to work as a medical representative. Responses ranged from 1 (very unimportant) to 5 (very important). All items were skewed toward 5 (very important), indicating that participants found all the factors—performing a socially important job, achieving high earnings, further career development, easiness of getting a job—to be important. The least important factor was academic performance, with a mean of 3.1.

**Table 2:** Distribution of contributing factors for choosing to work as a medical representative, ranging from 1 (very unimportant) to 5 (very important).

	**Distribution of responses (%)**					**Mean**	**SD**
	**1**	**2**	**3**	**4**	**5**		
The possibility of further career development	4.5	6.8	15.0	21.1	52.6	4.1	1.1
The prospect of performing socially important and interesting work	5.3	7.5	15.8	37.6	33.8	3.9	1.1
The ease of getting a job (saudisation of the sector)	11.0	18.0	33.0	31.0	40.0	3.5	1.2
The possibility of achieving high earning	6.0	6.0	18.0	31.6	38.3	3.9	1.6
Academic performance	8.3	13.5	24.8	23.3	30.1	3.1	1.1

Table 3 shows the distribution of scores of the seven perceptions regarding the medical representative career pathway. Responses ranged from 1 (strongly disagree) to 5 (strongly agree). Most items were skewed toward 5 (strongly agree): selling drugs through internal relationships, working under pressure, irregular career pathway, and sales oriented work rather than academic. The exceptions were the items concerning “the lack of a sense of honor and value” and “commercial bribery and sales commissions,” with a mean of 2.9 and 2.6, respectively.

**Table 3:** Distribution of perceptions about medical representative career pathway, from 1 (strongly disagree) to 5 (strongly agree).

	**Distribution of responses (%)**					**Mean**	**SD**
	**1**	**2**	**3**	**4**	**5**		
Medical representatives sell drugs through internal relationships and social networks	15	21.1	21.1	28.6	14.3	3	1.3
Medical representatives worked under high pressure, irregular career development and great mobility	4.5	7.5	18.8	39.8	29.3	3.8	1
The competition between pharmaceutical enterprises is fierce, and the laws and regulations are imperfect	12.8	15.0	30.1	23.3	18.8	3.2	1.3
Work is sales-oriented rather than academic based	7.5	15.0	19.5	25.6	32.3	3.6	1.3
Lacking the sense of honor and value, this industry is not attractive	21.8	18.0	21.8	21.1	17.3	2.9	1.4
The career orientation of medical representatives is unclear, and the management is not standardized	12.8	18.8	25.6	23.3	19.5	3.1	1.3
Commercial bribery and sales commissions are generally acceptable	31.6	12.8	26.3	16.5	12.8	2.6	1.4

The distribution of scores of 5 job-related factors is noted in Table 4. Responses ranged from 1 (strongly disagree) to 5 (strongly agree). All items were skewed toward 5 (strongly agree), indicating high job satisfaction, working in this sector long term, commitment toward working as medical representative, low intention to leave the sector, and would recommend working in this sector.

**Table 4:** Distribution of job satisfaction, work commitment, and intentions to leave ranging from 1 (strongly disagree) to 5 (strongly agree).

	**Distribution of responses (%)**					**Mean**	**SD**
	**1**	**2**	**3**	**4**	**5**		
I am satisfied with my current role in this sector	7.5	18.8	21.8	27.1	24.8	3.4	1.2
I see myself working in this sector long term	6.0	17.3	21.8	36.1	18.8	3.4	1.2
I am committed to working in this sector	5.3	12.8	18.0	39.8	24.1	3.6	1.1
I would recommend this career pathway for young pharmacists	3.0	6.8	21.1	34.6	34.6	3.9	1
I have intentions to leave this sector^*^	16.4	28.4	20.9	21.6	11.9	3.4	0.68

* Negatively worded questions. Direction reversed in subsequent analyses.

Table 5 presents the distribution of the variables being investigated. These scales are treated as continuous variables, ranging from 1 (strongly disagree) to 5 (strongly agree) for perceptions and job satisfaction, and 1 (very unimportant) to 5 (very important) for factors in choosing this pharmaceutical industry. The mean value of the overall scales—perceptions, job satisfaction and choice of industry—were 3.7, 3.2 and 3.4, respectively. All scales had a Cronbach alpha coefficient >0.7, indicating inter-item reliability.

**Table 5:** Distribution and internal consistency of overall scales.

**Description of scale**	**Distribution of responses (%)**					**Mean**	**SD**	**Cronbach alpha**
	**1**	**2**	**3**	**4**	**5**			
Factors for choosing to work in this sector	3.8	10.5	21.1	62.4	100	3.7	0.96	0.87
Perceptions about medical representative career pathway	1.5	9.0	48.1	84.2	100	3.2	0.88	0.806
Job satisfaction, work commitment, and intention to leave	0.8	8.3	30.1	66.2	100	3.4	0.67	0.84^*^

* Leaving excluded from the scale.

## Discussion

The current study was conducted to provide an overview of working as a medical representative in the pharmaceutical industry/pharmaceutical companies. The pharmaceutical industry as a pharmacy career choice was not among the top options for Saudi pharmacists in local studies conducted before 2018 (13, 14). According to the SCFHS, only 11.1% of the workforce in this sector were nationals in 2017 (6). With growing numbers of pharmacy colleges around the country and increasing numbers of pharmacy graduates, job opportunities in the most preferred sector, hospital pharmacy, became very limited, resulting in rising unemployment rates (5, 12, 26). Overcoming this issue necessitated the contribution of the private sector in creating employment opportunities in other settings, i.e., community pharmacy, which was discussed in previous studies (7, 12), and the pharmaceutical industry. The regulatory bodies in the country, represented by the SFDA and Ministry of Human Resources and Social Development, launched a labor reform initiative to renationalize the profession in both private sectors, and hence generate more employment for Saudi pharmacists.

The study indicated that work as a medical representative in a pharmaceutical company was attractive for recent male graduates (86.5%) holding a first degree in pharmacy (90.2%), and 62.4% had work experience of <5 years. Factors that pharmacists considered when choosing this pharmacy field included achieving high earning, preforming a socially important job, and the possibility of further career development. Similar factors were reported in recent studies assessing Saudi pharmacists' views toward working in community pharmacy (7), indicating that pharmaceutical companies are becoming an appealing career path that fulfills participants' career aspirations. This also explains the shift in career preference to include this setting, as noted in more recent national literature (7, 10–12). Academic performance was the least important reason for choosing a pharmaceutical company as an employer; this was also reported in previous international studies (4, 27).

The second part of the study investigated the common misconceptions about the field. According to the participants, although personal relationships and social networking are crucial for successful pharmaceutical promotion and marketing, they denied that commercial bribery is acceptable or that the field lacked honor or value. Contrary to local studies that reported free meals, non-educational gifts, drug samples for personal use, and financial support to attend educational activities (17, 18), these participants did not agree with these practices. The SFDA have issued the Saudi Code of Pharmaceutical Promotional Practices in the Kingdom of Saudi Arabia which might have increased the level of professional practice by defining the relationship between company representatives and physicians (28). Job-related factors, including a large workload, excessive travel, focus on sales rather than academic knowledge, and lack of standardized management were found to be the disadvantages of this job. Similar findings were noted in studies conducted in Bangladesh and India (15, 16).

The third part of the study investigated job satisfaction, work commitment, and intention to leave. These pharmacists were generally satisfied with their jobs, showed high work commitment, saw their job as long-term, had limited intention to leave and would recommend this career pathway to recent pharmacy graduates. These overall positive indicators showed that a job as a pharmaceutical company representative is a promising career path, especially for younger pharmacists, and would help increase employment rates. These findings were in alignment with a previous study conducted in Saudi Arabia on pharmacists working in various settings; hence, working in this sector is as satisfying as working in other sectors (22).

The supply side of the local workforce, pharmacy colleges, should focus on equipping students with the required knowledge, skills, and values to practice in pharmaceutical companies. Most of the pharmacy schools around the country have adopted clinically-focused Pharm D programs (26). However, the current demand has shifted toward community pharmacy and the pharmaceutical industry/pharmaceutical companies (5, 6). A needs-based education would, therefore, include more didactic courses, such as marketing, ethics, communication, pharmacoeconomics, and pharmacoepidemiology, as well as field training in those sectors to make sure that pharmacy students are ready to join the job market upon graduation. Additionally, career advisory services at pharmacy colleges should ensure students are aware that pharmaceutical companies have the highest employment rate.

The study has several limitations. The cross-sectional study design limited data collection to a single point of time, so changes over time were not assessed. Limited published literature, both nationally and internationally, about the medical representative job as a career pathway made comparisons and conclusions challenging. The questionnaire was sent to only five pharmaceutical companies; hence, the variety of the sample might have been affected. Self-assessment of pharmacists, in particular their opinions on work commitments and intentions to leave might not have been accurate. This study findings might be only generalizable only to other countries with similar social and cultural factors.

## Conclusion

The current study was conducted to provide a snapshot of Saudi pharmacists working as medical representatives. The top factors for choosing this career path were performing a socially important job, receiving a high salary, and having further career development, indicating that it is an appealing choice that fulfills pharmacists' career ambitions. Common misconceptions about medical representatives' work, such as offering commercial bribery and lack of honor and value, were found to be incorrect. Pharmacists showed high job satisfaction, high work commitment, and low turnover intentions, and would recommend this career choice for pharmacists. Saudising pharmaceutical companies would help to create more jobs for the increasing numbers of pharmacy graduates.

## Data availability statement

The original contributions presented in the study are included in the article/Supplementary material, further inquiries can be directed to the corresponding author.

## Ethics statement

The studies involving human participants were reviewed and approved by the Ethical Committee of the Scientific Research, King Khalid University ECM# 2021-5511. The patients/participants provided their written informed consent to participate in this study.

## Author contributions

Data curation, formal analysis, and supervision: DA. Funding acquisition, methodology, and project administration: DA and AA. Both authors contributed to the article and approved the submitted version.
