# Supplementary material for: A cross-sectional study on Saudi pharmacists working as medical representatives: What attracted them and what is keeping them in this sector—Misconceptions and reality
Source: Front Public Health. 2023 Mar 8;11:996536. doi: 10.3389/fpubh.2023.996536 (PMC10030677; doi:10.3389/fpubh.2023.996536)
Supplement: Supplementary file 1 [file Table_1.docx]

| Reviewer comment | Reply | Change |
| --- | --- | --- |
| 1. The questionnaire should be included in the manuscript as a figure for other groups to be able to utilize or replicate study | The questionnaire was added as an appendix |  |
| 2. Please include if the questionnaires where fully completed. If questionnaires were partially filled out were they still included? |  | There were no partially completed questionnaire as all questions were fully answered by respondents. |
| 3. The results section can include more details. Expanding on the results and what they can potentially mean. For example noting that most of the surveys were done by males - can this contribute to the differences? | More elaboration was added | Two thirds (66.9%) of the participants were young pharmacists between the ages of 23 and 30 years, and the majority (86.5%) were male which might indicate that this career pathway is a male-dominated pharmacy sector that attracts young professionals. The vast majority (90.2%) had achieved the first degree in pharmacy, while just under half (48.1%) had between 1 to 5 years work experience, which might mean that young pharmacists maybe see themselves working in this sector in the future. |
| 4. Has the data been analyzed by grouping based on age. Does the different age groups have different preferences/outcomes? | Majority of participants were younger pharmacist as this sector has recently been renationalised, only 1 pharmacist was between the age of 41-50 and none was older than 50, hence conducting regression wouldn’t have been valid. |  |
| 5. The limitations of the study can be more thoroughly addressed. Discussing how is this data applicable to the other populations or how the age groups or the fact that the submissions were mainly male could affect the data. | The nature of the job in this sector is believed to make it less attractive to female pharmacists. Although majority of participants were males, we believe that the sample truly represents the population. | This study findings might be only generalizable only to other countries with similar social and cultural factors. |
| 6. The manuscript needs to be proofread as their are some grammatical and sentence structure errors and addressing these will help with the flow of the manuscript. |  | The manuscript was proofread by a professional native speaker. |

|  |  |  |
| --- | --- | --- |
| The authors provide an interesting descriptive review on job satisfaction and work commitment for pharmacists who choose to work in the industry. The background information was adequate, the statistical plan, including sample size calculation was appropriate, and the results were presented well. I was impressed that the authors did not provide inferential statistics, but rather, focused on summary statistics. The summary statistics tell an interesting story. Additionally, the authors provide a measure of reliability for their project. | Thank you very much for your positive feedback |  |
| Maintain consistency with decimal places in the tables—use one or two, but verify all decimal places are consistent. For example, for the possibility of achieving high earnings individuals who replied with a one was given a 6 percent—should be 6.0 percent. | All decimal places were verified |  |

Data collection tool

Dear colleague,

We would like to invite you to take part in our study that aims to clarify misconception about working as medical representative, and to assess factors for choosing this career pathway as well as to evaluate job satisfaction. It will take around 10 minutes to complete. We appreciate your time and contribution

1. Age in years
2. 23-30
3. 31-40
4. 41-50
5. >50
6. Gender
7. Male
8. Female
9. Qualification
10. Bachelor degree
11. Postgraduate degree
12. Nationality
13. Saudi
14. Non Saudi
15. Work experience in years
16. <1
17. 1-5
18. >5
19. Factors for choosing to work in this sector -Please rate the following statements

|  | Very unimportant | Unimportant | Neutral | Important | Very important |
| --- | --- | --- | --- | --- | --- |
| The prospect of performing socially important and interesting work |  |  |  |  |  |
| The possibility of achieving high earning |  |  |  |  |  |
| The possibility of further career development |  |  |  |  |  |
| Academic Performance |  |  |  |  |  |
| The ease of getting a job (Saudization of the sector) |  |  |  |  |  |

1. Perceptions about medical representative career pathway- Please rate the following statements

|  | Strongly disagree | disagree | Neutral | Agree | Strongly agree |
| --- | --- | --- | --- | --- | --- |
| Medical representatives sell drugs through internal relationship and social network |  |  |  |  |  |
| Medical representatives worked under the high pressure, irregular career development and great mobility |  |  |  |  |  |
| The competition between pharmaceutical enterprises is fierce, and the laws and regulations are imperfect |  |  |  |  |  |
| Work is sales-oriented rather than academic based |  |  |  |  |  |
| Lack of the sense of honor and value, this industry is not attractive. |  |  |  |  |  |
| The career orientation of medical representatives is unclear, and the management is not standardized |  |  |  |  |  |
| Commercial bribery and sales commission are generally acceptable |  |  |  |  |  |

1. Job satisfaction, work commitment, and intentions to leave- Please rate the following statements

|  | Strongly disagree | disagree | Neutral | Agree | Strongly agree |
| --- | --- | --- | --- | --- | --- |
| I am satisfied with my current role in this sector |  |  |  |  |  |
| I see myself working in this sector long term |  |  |  |  |  |
| I am committed to working in this sector |  |  |  |  |  |
| I have intentions to leave this sector |  |  |  |  |  |
| I would recommend this career pathway for young pharmacists |  |  |  |  |  |
